# Media Access is Associated with Knowledge of Optimal Water, Sanitation and Hygiene Practices in Tanzania

**DOI:** 10.3390/ijerph16111963

**Published:** 2019-06-03

**Authors:** Chelsi C. Alexander, Shilpa Shrestha, Mamadou D. Tounkara, Shelly Cooper, Leiema Hunt, Taylor H. Hoj, Kirk Dearden, Dotto Kezakubi, Vianney Atugonza, Joshua West, Benjamin Crookston, Cougar Hall

**Affiliations:** 1 Department of Public Health, Brigham Young University, Provo, UT 84602, USA; chelsicalexander@gmail.com (C.C.A.); ceelpa.ss@gmail.com (S.S.); souckos3@gmail.com (M.D.T.); shelly@rockymountaininstall.com (S.C.); leiema.hunt@gmail.com (L.H.); taylorhoj@gmail.com (T.H.H.); josh.west@byu.edu (J.W.); benjamin_crookston@byu.edu (B.C.); 2 IMA World Health, Nyalali Curve, Dar es Salaam, Tanzania; kirkdearden@imaworldhealth.org; 3 DMI Tanzania, P.O. Box 517, Mwanza, Tanzania; dotto.kezakubi@developmentmedia.net (D.K.); vianney.atugonza@developmentmedia.net (V.A.)

**Keywords:** water, sanitation, hygiene, media access, Tanzania

## Abstract

The importance of water, sanitation and hygiene (WASH) behaviors in low- and middle-income countries in preventing childhood illness is well established. Tanzania is known to have high rates of chronic malnutrition and childhood stunting—both of which have been linked to poor WASH practices. Interviews were conducted with 5000 primary caregivers of children aged 0–23 months. Four composite WASH knowledge variables were created to assess the relationship between WASH knowledge and access to different forms of media, such as television, radio, and mobile phones. WASH knowledge variables measure knowledge of when to wash hands, the need for soap when washing hands, when to wash a baby’s hands, and how eating soil or chicken feces can affect a baby’s health. Logistic and linear regression analyses were conducted to measure the association between media access and WASH knowledge. Having watched television was positively associated with higher WASH knowledge indicators (all *p* < 0.05). Higher WASH knowledge was positively associated with more frequent handwashing after cleaning a baby’s bottom (all *p* < 0.0001). The quantity of media access also had a positive linear effect on handwashing; more media items owned was associated with increases in handwashing. Study findings indicate media access is associated with WASH knowledge among caregivers in resource-poor settings.

## 1. Introduction

The importance of water, sanitation and hygiene (WASH) behaviors in low- and middle-income countries to child health and development and household health is well established [1,2,3,4,5,6]. Much is known about how WASH interventions can be used to increase WASH knowledge and support behavior change that will lead to improved health outcomes, such as reducing stunting rates [1,2,3,4,5,6]. The prevalence of stunting in developing countries, including those in sub-Saharan Africa and Southeast Asia, is among the highest in the world due to numerous known factors—among these are a lack of appropriate education and the low availability of hygiene facilities and resources, such as soap [1,2,3,4,5,6].

WASH interventions during early childhood promote healthy behaviors, decrease the occurrence of conditions such as enteropathy and stunting, and reduce child mortality rates. The period of early childhood is a crucial stage of development that can affect the health status of individuals for the remainder of their lives. Research has shown that WASH practices during this period (the first 3 years of life) directly impact health, as well as cognitive and physical development [7,8,9]. Environmental enteropathy may result from poor WASH practices and a general lack of cleanliness [7]. Enteropathy prevention efforts include increasing access to clean water and improved sanitation facilities as well as promotion of proper handwashing behaviors [10,11].

Tanzania has some of the highest known rates of chronic malnutrition and childhood stunting [12]. Malnutrition and stunting in Tanzania have been linked to poor WASH practices. An estimated 40% of Tanzanians currently get their drinking water from an unimproved source [13]. A 2016 report noted that efforts to improve water sources were lacking in effectiveness as 40% of water points were reported as non-functional and many were failing within the first year after construction [14]. Additionally, most Tanzanians practice fixed-point defecation and 80% rely on basic unimproved sanitation facilities [13]. Up to one third of deaths in children under-five years in sub-Saharan Africa are related to poor hygiene, with the bulk of these deaths due to preventable diarrhea [15]. Inadequate access to clean water sources, poor sanitation, and poor hygiene behaviors in Tanzania contribute to the occurrence of diarrhea exacerbating stunting and malnutrition.

Funded by the United Kingdom’s Department for International Development (DFID) and implemented by IMA (Interchurch Medical Assistance) World Health, the Addressing Stunting in Tanzania Early (ASTUTE) program addresses improving WASH practices for caregivers of children aged two and younger in Tanzania. ASTUTE aims to increase knowledge related to stunting prevention behaviors among pregnant women, caregivers, households, and community decision makers. As part of this project, a baseline survey was developed by Development Media International (DMI), a partner of IMA World Health. In addition to providing key baseline measures for monitoring and evaluation efforts, survey results have informed the design of key ASTUTE programmatic elements and strategies. Using ASTUTE baseline data, this study examines the association between media access and knowledge of appropriate WASH practices in Tanzania.

## 2. Materials and Methods

### 2.1. Design

Data were collected in 2016 and come from the baseline survey for the ASTUTE intervention in Tanzania’s Lake Zone, home to approximately 10.5 million people. Districts were selected using probability proportional to size sampling with a sampling frame from Tanzania’s most recent census. Wards, villages, and streets were chosen randomly from the selected districts. After the districts were selected, wards within each selected district were then randomly selected, followed by random selection of villages/streets. Enumerators then systematically visited households until the required number of interviews with eligible participants had been completed.

### 2.2. Sample

Eligible participants included primary caregivers with a child aged 0–23 months. 5000 primary caregivers were interviewed. Participant demographics are presented in Table 1. 

### 2.3. Procedure

Authorization to conduct the research and recruit study participants was obtained from local authorities, including Regional Medical Officers (RMOs) and/or District Medical Officers (DMOs). Ethical clearance was obtained from the National Institute for Medical Research in Tanzania (NIMR/HQ/R.8a/Vol. IX/2344). After enumerator training, the instrument was pilot-tested and finalized before being uploaded onto Android mobile devices used for data collection. The survey team (IPSOS Tanzania) then traveled to randomly selected wards and villages or streets to administer the survey. Within each village, the team began the survey with the assistance of the Village Executive Officer or another village/street guide who introduced the survey team to households. Interviews were conducted in Kiswahili. A maximum of three attempts were made to visit households before they were substituted with replacement households. 

### 2.4. Measurement and Analysis

Participants reported demographic information related to age, religion, ethnicity, marital status, and educational attainment. Socioeconomic status was determined via an asset index, which was calculated by summing the total number of household assets owned from 21 items. Assets included a bicycle, motorcycle, car or truck, animal-drawn cart, boat with motor, boat without motor, radio, television, mobile phone, refrigerator, table, chairs, bed, air conditioner, computer, electric iron, fan, power tiller, connection to the national electricity grid, active mobile banking account, and owning more than one acre of agricultural land. 

Four indicators measuring WASH knowledge were created using the following questionnaire items: “please mention all the occasions when it is important to wash your hands” (labelled handwashing/when to wash hands; correct answers included: after latrine use; after assisting a child who has defecated; before preparing food; before eating food; before feeding a child; after cleaning the compound; after contact with animal feces), “does handwashing with water alone make your hands clean?” (labelled water only/need for soap when washing hands; correct answer: yes), “when should you wash a baby’s hands? Please tell me all your ideas” (labelled washing baby’s hands/when to wash baby’s hands; correct answers included: after a nappy change or toilet use; after playing in the yard; before feeding/eating time; when hands are visibly dirty), and “according to you, how can eating soil or chicken poop affect a baby’s health? Please tell me all your ideas” (labelled eating soil or chicken feces/child eating soil; correct answers included: makes baby grow poorly; makes baby’s brain develop poorly; causes stomach ache; causes diarrhea/illness; causes worm). Participants were given a point for each correct response indicating knowledge and no points for incorrect responses or responses coded “other”. These variables had possible scores of 0–7, 0–1, 0–4, and 0–5 respectively, with 0 indicating no knowledge and the upper limit indicating the highest measurable knowledge for each variable. 

Media access was measured by the following questions: “if respondent owns a radio: does the radio in your household have batteries, is it currently working?”, “when did you last listen to the radio?”, “when did you last watch the television?”, “do you personally own a mobile phone?”, “do you have WhatsApp on your mobile phone?”, and “does anyone in your household own a mobile phone?”. Logistic and linear regression analyses were conducted to measure the association between media access and WASH knowledge. Unadjusted and adjusted models were computed. Adjusted models included age, education level, and number of household assets. Religion and ethnicity were dropped from the models as they were statistically insignificant and, in the case of ethnicity, had limited statistical power due to insufficient diversity. All analyses were conducted using SAS^®^ version 9.4 (SAS Institute Inc., Cary, NC, USA).

## 3. Results

Respondent demographics are presented in Table 1. Of the 5000 respondents, 50.94% were 20 to 29 years old. Nineteen-percent of respondents had no formal education, while 68.02% had attended or completed primary school. Respondents could report a maximum of 21 household assets; the highest number of assets reported was 15 and the mean number of assets was 4.8 (standard deviation [SD] 2.0). 

The normal distribution of the four study variables measuring WASH knowledge are presented in Table 2. The “water only” variable results are the product of logistical regression analysis, while results for the other three study variables are the product of linear regression modeling. 

### Media Access and WASH Knowledge

Based on unadjusted models, ‘*having a working radio*’ and ‘*owning a house phone*’ were positively associated with higher WASH knowledge scores for each of the composite WASH knowledge variables. This association did not remain significant in the adjusted models. ‘*Having someone in the household who owned a mobile phone*’ was associated with an increase in knowledge of ‘*when to wash hands, need for soap when washing hands*’ and ‘*when to wash a baby’s hands*’ for the unadjusted models; this association was only significant for ‘*need for soap when washing hands*’ in the adjusted models. When the respondent ‘*last reported listening to the radio*’ was only significant in one instance: having listening yesterday was significant for ‘*when to wash baby’s hands*.’ 

The following associations were significant for both the unadjusted and adjusted models. ‘*Having watched television*’ was positively associated with an increase in WASH knowledge for each of the composite WASH knowledge variables. In each case, the association was strengthened when the respondent reported ‘*having watched television in the last month*.’ ‘*Having WhatsApp on a mobile device*’ was also associated with an increase in the knowledge of ‘*when to wash hands* and *when to wash a baby’s hands*.’ These results are presented in Table 3, Table 4, Table 5 and Table 6.

## 4. Discussion

This study sought to understand the association between media access and WASH knowledge. 

### 4.1. Using Television for Health Education

The positive association of media access and WASH knowledge in the current study lends support to the continued use of mass media for health interventions. A study conducted in Kenya measured the relationship of media ownership and influence with handwashing. Media ownership included television, radio, email address, and postal address. Media exposure included newspapers, radio, television, and movie. Exposure was measured over a month. Consistent with the current study, results in Kenya showed a positive correlation to media access and an increase in handwashing. The amount of media ownership also had a positive linear effect on handwashing; the more media items owned equated to the increase in handwashing [16]. A cross-sectional study in Bangladesh similarly identified a strong association between television access and handwashing practices [17].

Beyond mere access, the use of mass media for health education and to promote health knowledge and behavior has been well documented [18,19,20]. In their systematic review of the literature on mass media interventions targeting child health, Naugle and Hornick include interventions targeting diarrheal disease, immunization, malaria, nutrition, HIV, respiratory disease, and reproductive health [18]. Radio is the most frequently used channel followed by television in these interventions [18]. Radio and television have been used to specifically increase WASH knowledge and behaviors previously among targeted populations. A national handwashing campaign took place in Ghana in 2003–2004 [21]. The campaign utilized a combination of mass media and community events to reach 82% of the population. Television and radio had a greater influence than community events but combined there was a 30% increase in WASH practices. It is important to note that television had a greater impact on awareness than radio campaigns. Television ads were run at peak times on three different channels. Exposure to both television and radio did not enhance the results [21]. 

### 4.2. Benefits of WhatsApp

The data presented here demonstrate a positive correlation between the use of WhatsApp on a mobile device and some aspects of WASH knowledge. WhatsApp has become a common communication medium in developing settings and several studies have concluded that it is suitable for health applications in low-resource settings [22,23,24,25]. According to Siemens, knowledge resides in the network and might be leveraged for the health of the public with communication applications designed for knowledge sharing [26]. WhatsApp appears to have become a networking tool capable of amplifying learning and making knowledge widely available as the network of users, tools, and connections are strengthened [27].

### 4.3. Planning Media Campaigns to Target Health Education 

Challenges related to planning media campaigns for health education in developing settings remain due to a variety of factors including limited or irregular access to electricity and underdeveloped infrastructures [21]. Radio interventions have the greatest reach, even in locales with low access to electricity, and tend to be the most common channel used [18]. No one channel or approach reaches everyone, however, so combining mass media with traditional health education approaches is strongly recommended. Efforts in Ghana combined mass media communication with community-sponsored events to promote appropriate handwashing practices [21]. The campaign resulted in a 30% increase in reported handwashing with soap after visiting the toilet or cleaning a child’s bottom. While television and radio had the greatest influence on self-reported handwashing, pairing these mass media channels with community events resulted in improved behavioral outcomes. The authors noted a continued need to provide integrated programs inclusive of community health education for those not reached by radio and television [21]. 

### 4.4. Health Behavior Theories Supporting Media Education 

The Theory of Planned Behavior posits that the best predictor of behavior change is an individual’s intention to perform the behavior. Intention, in turn, is determined by three factors: attitude toward the behavior, subjective norms, and perceived behavioral control [28]. Knowledge, while not always a strong predictor of behavior independently, is an effective tool in shaping behavioral beliefs and attitudes. Subjective norms have been shown to directly impact behavioral intention. Paraveen and colleagues identified social norms that impact the attitudes of young mothers living in rural Bangladesh toward handwashing [29]. This qualitative study concluded that health messages designed to promote WASH behaviors should be focused, simple, and inclusive of family and community members who help to establish normative behaviors [29]. In a meta-analysis conducted by Albarracin and colleagues, it was suggested that focusing on increasing positive attitudes and self-efficacy toward a behavior facilitates change better than messages that focus on the threat of a disease [30]. Tanzanian efforts to influence attitudes through increased WASH knowledge, illuminate subjective norms related to expectations for health-promoting WASH practices, and building self-efficacy for such practices may benefit from continued investment in family-oriented communication efforts and positive health messages related to WASH. 

### 4.5. Limitations

The results of this study should be considered in light of several key limitations. Data analyzed for this study did not include all aspects of WASH knowledge or all points of media access. The survey questionnaire was used to gather baseline data prior to media campaigns and programming implemented by IMA World Health, and was not designed specifically to address this study’s research question. This study focused on the association of media access rather than investigating how a specific and intentional media campaign was targeting WASH knowledge. Likewise, this study was limited in that it was only able to measure the association between media access and WASH knowledge, not actual WASH behaviors or behavioral intentions. Several key measures of media access were dependent on participant recall of over a month, potentially decreasing reliability of responses. In addition, because this survey collected only baseline data, some of the variables needed to construct more complex asset indices were not available, resulting in a simple asset index which measured the number of assets owned. The asset index used in this study was thus limited and may have resulted in errors related to unmeasured socioeconomic indicators. Future research is needed to determine the impact of specific WASH media messages on WASH knowledge and behaviors. 

## 5. Conclusions

Findings from this study suggest that various forms of media access are associated with WASH knowledge among Tanzanian caregivers. Being able to watch the TV lately reflected better knowledge scores on all study variables. Having a mobile phone and using WhatsApp were likewise associated with WASH knowledge. Media exposure may therefore play an important role in health promotion and education in developing countries. Many underserved populations still have limited access to current health information and health education. Communication channels such as television, radio, and the emergence of widely accessible mobile applications like WhatsApp, present tremendous opportunities for health promotion.

## Figures and Tables

**Table 1:** Participant demographics, *n* = 5000.

Demographics	*N* (%)
Age of primary female caregiver	
15–19	500 (10.00%)
20–29	2547 (50.94%)
30–39	1286 (25.72%)
40+	312 (6.24%)
*Missing age*	355 (7.10%)
Education status	
No formal education	953 (19.06%)
Attended primary	3401 (68.02%)
Attended secondary	596 (11.92%)
High school or greater	50 (1.00%)
Number of assets	
0–2	744 (14.88%)
3–5	2444 (48.88%)
6–8	1601 (32.02%)
9+	211 (4.22%)
Owns a radio	2374 (47.48%)
Owns a TV	585 (11.70%)
Has WhatsApp on mobile phone	214 (4.28%)

**Table 2:** Water, sanitation, hygiene (WASH) knowledge.

Variables	Handwashing *	Water Only **	Washing Baby’s Hands *	Eating Soil or Chicken Feces *
	*N* (%)	*N* (%)	*N* (%)	*N* (%)
	Mean = 2.9 (SD 1.54)	Mean = 0.87 (SD 0.13)	Mean = 1.5 (SD 1.00)	Mean = 1.05 (SD 1.02)
0	225 (4.50%)	622 (13.08%)	684 (13.68%)	1916 (38.32%)
1	567 (11.34%)	4346 (86.28%)	2014 (40.28%)	1468 (29.36%)
2	1349 (26.98%)		1484 (29.68%)	1084 (21.68%)
3	1307 (26.14%)		617 (12.34%)	516 (10.32%)
4	827 (16.54%)		201 (4.02%)	14 (0.28%)
5	416 (8.32%)			2 (0.04%)
6	162 (3.24%)			
7	147 (2.94%)			

* Linear Regression Model; ** Logistic Regression Model. SD = standard deviation.

**Table 3:** When to wash hands knowledge indicators associated with media access.

	Unadjusted Estimate (CI)	Adjusted Estimate (CI)
**When last listened to radio**		
1-More than a month ago	0.283 (−0.591–1.158)	0.356 (−0.538–1.251)
2-In the last month	0.486 (−0.402–1.376)	0.587 (−0.325–1.50)
3-In the last week	0.612 (−0.223–1.447)	0.717 (−0.138–1.573)
4-Yesterday	0.696 (−0.132–1.525) ***	0.716 (−0.535–0.897)
5-Today	0.701 (−0.122–1.525) ***	0.703 (−0.142–1.548)
**When last watched television**		
1-More than a month ago	0.463 (0.353–0.573) **	0.279 (0.163–0.395) ***
2-In the last month	0.649 (0.488–0.810) **	0.411 (0.245–0.577) ***
3-In the last week	0.668 (0.507–0.829) ***	0.366 (0.197–0.535) ***
4-Yesterday	0.716 (0.535–0.897) ***	0.215 (0.023–0.408) *
5-Today	0.846 (0.649–1.041) ***	0.234 (0.020–0.447) *
**Having WhatsApp on a mobile device**		
	0.693 (0.483–0.903) ***	0.455 (0.234–0.676) ***

Linear regression model; Reference group = no/never; CI = confidence interval; * *p* < 0.05; ** *p* < 0.001; *** *p* < 0.0001.

**Table 4:** Need for soap when washing hands knowledge indicators associated with media access.

	Unadjusted OR (95% Wald Confidence Interval)	Adjusted OR (95% Wald Confidence Interval)
**When last listened to radio**		
1-More than a month ago	Too few respondents	Too few respondents
2-In the last month	1.022 (0.513–2.038)	1.038 (0.513–2.104)
3-In the last week	0.892 (0.398–1.999)	1.001 (0.440–2.273)
4-Yesterday	1.189 (0.795–1.777)	1.120 (0.730–1.718)
5-Today	0.780 (0.530–1.148)	0.826 (0.549–1.244)
**When last watched television**		
1-More than a month ago	1.784 (1.460–2.179) *	1.593 (1.281–1.982) *
2-In the last month	2.907 (2.016–4.193) **	2.497 (1.700–3.666) **
3-In the last week	1.819 (1.327–2.493) *	1.607 (1.138–2.268) *
4-Yesterday	3.743 (2.351–5.959) ***	2.696 (1.660–4.380) **
5-Today	3.362 (2.064–5.477) ***	2.258 (1.344–3.795) **
**Having WhatsApp on a mobile device**		
	1.814 (0.993–3.315) *	1.383 (0.741–2.581) *

Logistic regression model; Reference group = no/never; OR = odds ratio; * *p* < 0.05; ** *p* < 0.001; *** *p* < 0.0001.

**Table 5:** When to wash baby’s hands knowledge indicators associated with media access.

	Unadjusted Estimate (CI)	Adjusted Estimate (CI)
**When last listened to radio**		
1-More than a month ago	0.238 (−0.334–0.811)	0.284 (−0.306–0.875)
2-In the last month	0.186 (−0.397–0.769)	0.241 (−0.361–0.844)
3-In the last week	0.355 (−0.191–0.903)	0.42 (−0.144–0.985)
4-Yesterday	0.561 (0.018–1.104) *	0.597 (0.035–1.159) *
5-Today	0.503 (−0.036–1.043)	0.54 (−0.017–1.098)
**When last watched television**		
1-More than a month ago	0.413 (0.342–0.484) ***	0.369 (0.293–0.445) ***
2-In the last month	0.663 (0.559–0.766) ***	0.617 (0.508–0.725) ***
3-In the last week	0.633 (0.529–0.737) ***	0.574 (0.463–0.685) ***
4-Yesterday	0.535 (0.418–0.652) ***	0.445 (0.319–0.571) ***
5-Today	0.483 (0.357–0.610) ***	0.370 (0.230–0.510) ***
**Having WhatsApp on a mobile device**		
	0.409 (0.269–0.549) ***	0.424 (0.275–0.574) ***

Linear regression model; Reference group = no/never; CI = confidence interval; * *p* < 0.05; *** *p* < 0.0001.

**Table 6:** Child eating soil knowledge indicators associated with media access.

	Unadjusted Estimate (CI)	Adjusted Estimate (CI)
**When last listened to radio**		
1-More than a month ago	0.142 (−0.456–0.740)	0.142 (−0.475–0.756)
2-In the last month	0.319 (−0.289–0.928)	0.313 (−0.317–0.943)
3-In the last week	0.289 (−0.282–0.860)	0.294 (−0.296–0.886)
4-Yesterday	0.394 (−0.172–0.9610)	0.383 (−0.203–0.97)
5-Today	0.411 (−0.152–0.974)	0.383 (−0.200–0.031)
**When last watched television**		
1-More than a month ago	0.256 (0.183–0.330) ***	0.172 (0.093–0.250) ***
2-In the last month	0.449 (0.341–0.556) ***	0.378 (0.266–0.491) ***
3-In the last week	0.291 (0.183–0.398) ***	0.186 (0.072–0.301) *
4-Yesterday	0.304 (0.182–0.425) ***	0.165 (0.035–0.296) *
5-Today	0.342 (0.211–0.473) ***	0.170 (0.025–0.315) *
**Having WhatsApp on a mobile device**		
	0.145 (−0.001–0.292)	0.056 (0.098–0.210)

Linear regression model; Reference group = no/never; CI = confidence interval; * *p* < 0.05; *** *p* < 0.0001.
